# Program Report: Implementing Mental Health Supports and Resources for Dialysis Patients Into Practice Through the Mind the Gap Project

**DOI:** 10.1177/20543581261487730

**Published:** 2026-09-16

**Authors:** Kara Schick-Makaroff, Charlotte Berendonk, Ashley Seitz, Rommelson Aquino, Mark Canney, Joel Davidson, Reena Davis, Arlene Desjarlais, Priscila Ferreira da Silva, Denise Fillier, Owen Gaskill, Earl Halcrow, Oksana Harasemiw, Dawn Hau, Lisa Joya, Joanne Kappel, Scott Klarenbach, Jonathan Lee, Loretta Lee, Russell Malabanan, Michael McCormick, Primrose Mharapara, Frances Reintjes, Richard Sawatzky, Karthik Tennankore, Stephanie Thompson, Lauren Virostek, Clara Bohm

**Affiliations:** 1 Faculty of Nursing, College of Health Sciences, 3158 University of Alberta, Edmonton, AB, Canada; 2 Max Rady College of Medicine, 8664 University of Manitoba, Winnipeg, MB, Canada; 3 Department of Medicine, University of Ottawa, Ottawa, ON, Canada; 4 Patient partner with Can-SOLVE-CKD Network, Canada; 5 Rady Faculty of Health Sciences, 8664 University of Manitoba, Winnipeg, MB, Canada; 6 Alberta Kidney Care South, 3146 Alberta Health Services, Calgary, AB, Canada; 7 Chronic Disease Innovation Centre, Seven Oaks General Hospital, 8664 University of Manitoba, Winnipeg, MB, Canada; 8 432234 Nova Scotia Health Renal Program, Nova Scotia Health Authority, Halifax, NS, Canada; 9 7235 Department of Medicine, University of Saskatchewan, Saskatoon, SK, Canada; 10 Faculty of Medicine, College of Health Sciences, 3158 University of Alberta, Edmonton, AB, Canada; 11 Alberta Kidney Care North, 3146 Alberta Health Services, Edmonton, AB, Canada; 12 School of Nursing, 4402 Trinity Western University, Langley, BC, Canada; 13 Centre for Advancing Health Outcomes, Providence Health Care Research Institute, Vancouver, BC, Canada; 14 Sahlgrenska Academy & Centre for Person-centred Care, University of Gothenburg, Gothenburg, Sweden; 15 Division of Nephrology, Department of Medicine, Dalhousie University, Halifax, NS, Canada

**Keywords:** mental health, implementation, dialysis, patient-oriented research

## Abstract

**Purpose of Program:**

People receiving dialysis experience a high burden of mental health challenges. Few dialysis-specific resources exist to support their mental well-being. The Can-SOLVE CKD Network ‘Mind the Gap’ project team identified mental health care challenges for the dialysis population and explored strategies to integrate relevant support and resources into practice.

**Sources of Information:**

Candidate interventions were a Dialectical Behavioural Therapy (DBT) Skills Training program implemented for people receiving hemodialysis in Manitoba and a provincial pathway for mental health care for people receiving all dialysis modalities at a system-level in Alberta. For this reason, we refer to both hemodialysis and dialysis in this report.

**Methods:**

Mind the Gap’s two interventions were identified after input from diverse interest-holders including people with lived dialysis experience, their caregivers, and kidney and mental health care providers and leaders. Emphasis was placed on patient engagement, Equity, Diversity, Inclusion and Accessibility (EDI-A), and Indigenous cultural safety. The Knowledge to Action Framework informed implementation.

**Key Findings:**

Looking across both interventions, at the person-level, barriers included stigma around asking about and accessing mental health resources, and the perspective that mental health care does not belong in dialysis care. To empower people on dialysis to advocate for their mental health needs, we developed resource lists of freely accessible, online mental health supports for Canadians receiving dialysis. From the DBT project, Indigenous patients and patient partners were more engaged when the Mind the Gap Indigenous Research Coordinator was involved in team interactions. At the healthcare provider-level, the importance of mental health care in dialysis populations was recognized but there was uncertainty as to who was responsible to address this. Champions in various roles, including patient partners, healthcare providers, and leaders, have been a constant strategic facilitator. Intervention-level facilitators for DBT included holding DBT sessions at a location away from the hemodialysis unit and obtaining funding for allied healthcare providers to complete DBT Skills training. Intervention-level facilitators for the provincial pathway for mental health care for people receiving dialysis included engaging hundreds of knowledge users in its development. At both the intervention and system-levels, barriers included the impact of interventions on clinical care resources or workflow and high turnover of program leadership.

**Limitations:**

Our work reflects the perspectives of interest-holders in two Canadian provinces. Transferability of findings about mental healthcare challenges, available supports, and interventions to diverse dialysis populations across Canadian contexts requires further investigation.

**Conclusion:**

All interest-holders expressed a compelling call to attend to mental health support needs in dialysis patients. The process involves numerous challenges, including stigma associated with people receiving dialysis accessing and healthcare providers engagement in mental health supports, time constraints, lack of relevant training among dialysis staff, and inflexible, siloed structures within healthcare systems. Blending mental health care with dialysis care is culturally novel and an important step to meaningfully support the emotional and mental well-being of people on dialysis.

## Introduction

Living with kidney failure and receiving dialysis treatment comes with physical and mental challenges. As people transition to dialysis they experience abrupt changes to their life including: the need to reallocate significant portions of time each week for dialysis, loss of independence and normalcy, increased fatigue, inability to do pre-dialysis activities, and restrictive diets. It is therefore not surprising that people receiving dialysis report mental health challenges at disproportionately high rates. Four percent of the general population report symptoms of depression. However, this proportion increases to 20-40% in people receiving dialysis.^1-4^ People receiving dialysis who report mental health challenges have more frequent emergency department visits,^5^ greater risk for hospitalization,^6,7^ longer hospitalizations,^8^ increased mortality,^9,10^ lower odds of kidney transplantation,^11^ and reduced quality of life compared to those receiving dialysis without mental health challenges. While these health outcomes are documented, access to relevant mental health supports and resources has been identified as a care gap.^3,12,13^

Our team of researchers, administrators, and patient partners with lived dialysis experience is working to close this gap and improve person-centered kidney care through the patient-oriented research (POR) network, Canadians Seeking Solutions and Innovations to Overcome Chronic Kidney Disease (Can-SOLVE CKD).^14^

In June 2019, we held a priority-setting workshop involving multiple interest-holders with facility-based dialysis experience from across Canada as part of an initial Can-SOLVE CKD project called ‘Information, Interaction and Individualization - “Triple I”: Improving Facility-Based Hemodialysis Care.’^13^ Through our workshop processes, we identified better access to mental health supports and programs as a top priority. We officially launched the ‘Mind the Gap’ project in 2022 to identify key challenges and priorities for mental health in people receiving hemodialysis and to inform the implementation of person- and system-level mental health interventions. (See Patient Partner Reflection, Quote 1, Table 1 ). The objectives for the project were:

1. Develop an accessible inventory of mental health supports available to Canadians on all dialysis modalities by performing an environmental scan of freely-accessible mental health resources.^15^
2. Identify culturally sensitive, individualized approaches to mental health care for people on hemodialysis by identifying priority challenges to mental health and preferences for mental health support strategies for Canadians on hemodialysis.^16,17^
3a. Identify, develop, and implement an intervention to support mental health care for individuals receiving hemodialysis in Manitoba.
3b. Implement a pathway for mental health care for Albertans receiving any dialysis modality.

**Table 1.** Patient Partner Reflections

Quote #	Patient partner	Reflection
Quote 1	Patient receiving hemodialysis	*“Mental health can deteriorate in a split second and linger long after. If a patient has no one to talk to, it can become worse than the disease.”*
Quote 2	RD, DBT team member	*“Along with being a therapist, I have chronic kidney failure and underwent a kidney transplant in 2018. As I reflect on my time on dialysis and before that, I know that there were many times that I used mindfulness to adapt to worsening kidney function, to the fear of what came next for me and the anger that I experienced at times. It felt unfair sometimes and I would fight against it, which didn’t help at all. It left me frustrated because there was nothing I could do to change what was - there’s no cure for this disease. Sure, I kept a healthy lifestyle, followed advice on what to eat, taught fitness and yoga. But none of that mattered. The disease is still there. Through some of the techniques that I now teach to others, I was better able to manage those feelings. Not ignore them; to feel them fully and get to the other side. The techniques in DBT are very practical. They don’t rely on introspection alone, which makes them easy to use with a mind that won’t still. When one (skill) doesn’t work, I can move to a different skill. That, for me, is the power behind DBT.*
		*Through my journey with kidney disease and dialysis, I have felt frustrated by loved ones who try but don’t quite understand what I’ve felt. Interpersonal skills are very useful at these times. Other times, my emotions have been all over the place from fear to depression to hope. Emotional regulation is needed in these times. Other times I have felt distressed at what I might offer as a parent or a partner or even colleague. Distress tolerance skills are needed here. And behind it all I have practiced mindfulness to stay as present as possible.*
		*DBT doesn’t mean never experiencing these feelings, it’s about feeling them but building the resilience to come out of it more quickly than before.”*
Quote 3	LL, Community Advisory Committee member	*“As a patient partner, it is very meaningful to be on the ‘ground floor’ of building a mental health pathway. Giving feedback on wording and giving the patient perspective on the many aspects of building a pathway has been an interesting process, and it’s not over yet.”*
Quote 4	Patient receiving dialysis	*“I think it would have been nice to hear someone say, ‘What’s going on? Why are you depressed or upset?’ And someone that can show that they care, not just put the physical part, but the emotional, I would love to have someone to say, ‘Tell me what’s going on, or why, today, you feel sad….’ But on that part, when it comes to mental illness, they don’t really focus on that. My guess: they have too many patients to deal with. But it would have been great to see someone said, ‘You know what? Let’s take another 10 minutes to find out what’s going on and why you feel that way.’ I think that would have been great.”*
Quote 5	JC, Community Advisory Committee member	*“When I was first diagnosed with kidney disease, I was basically on my own and had to figure out things on my own with the help of a part-time social worker. I tried group counseling and that wasn’t the right fit for me. Eventually, after a long wait, I got a referral from my family doctor to see a psychologist and that has been really helpful. I know the social workers at the dialysis units are spread pretty thin and that’s when the other healthcare workers need to step in. It’s part of their job. Sometimes all you really need is just someone to talk to, right.”*
Quote 6	JC, Community Advisory Committee member	*“I hope that information about mental health will become more accessible and that some of the stigma around mental health can be erased. It would be great to see people use this pathway and for it to become commonplace in the kidney care setting.”*

In this program report, we focus on Objectives 3a and 3b. We describe the steps taken to address mental health through two interventions, one at a person-level: a Dialectical Behavioural Therapy (DBT) Skills Training program; and one at a systems-level: a provincial pathway for mental health care for people receiving dialysis. We highlight key learnings gleaned from our knowledge mobilization journey to translate findings into practice (Figure 1).

**Figure 1.**
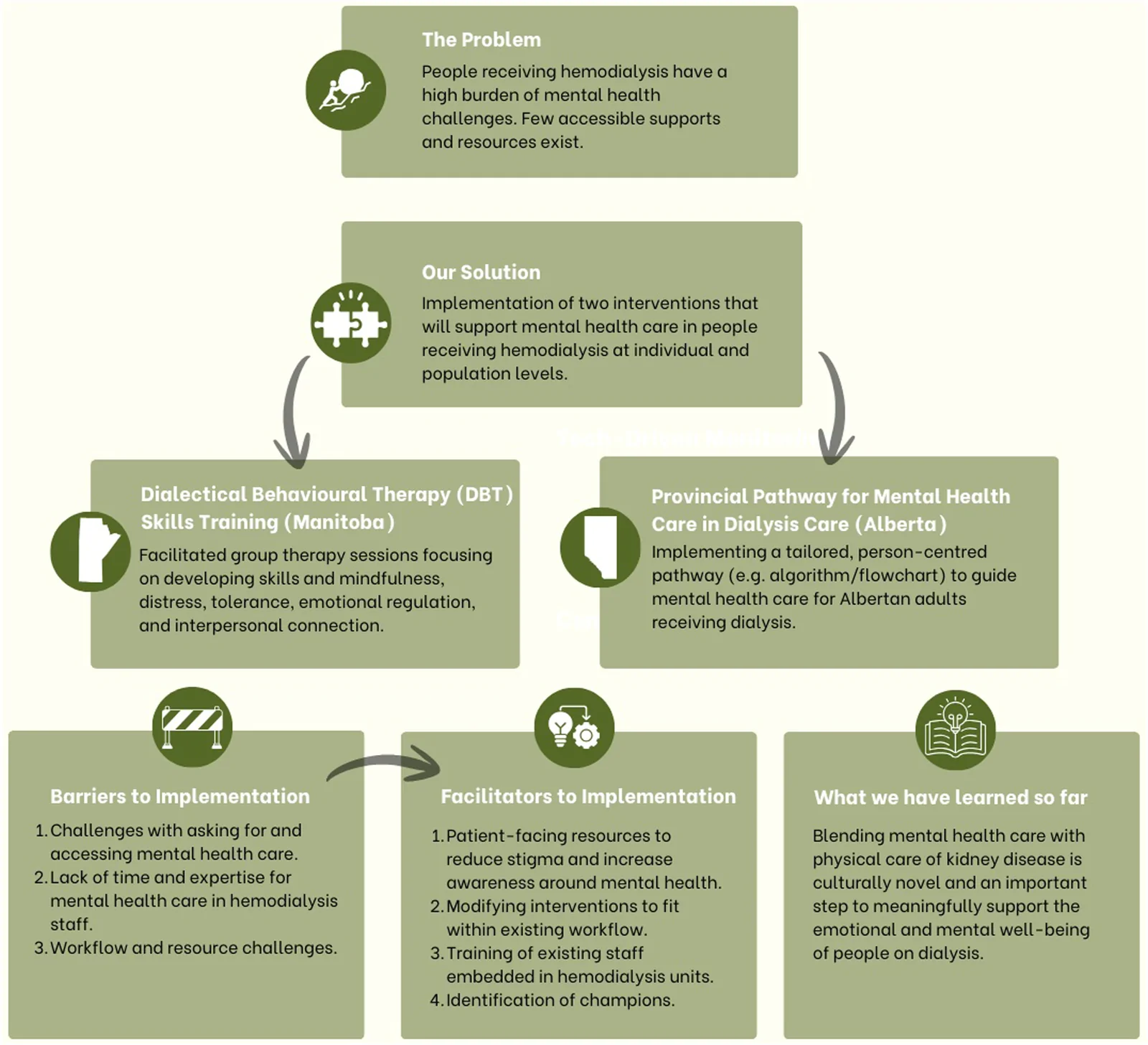
Mind the Gap Project implementation overview

## Objective 3a: Dialectical Behavioural Therapy Skills Training Program for People Receiving Hemodialysis

### Identifying an Appropriate Intervention

We performed a cross-sectional national survey gathering perspectives on mental health in hemodialysis from people receiving hemodialysis, their caregivers, and dialysis healthcare providers that identified a shortlist of 25 common challenges to mental health. Subsequently, a 3-day priority-setting workshop with diverse interest-holders was held in June 2024. We used the shortlist of challenges and consensus methodology to identify the top five challenges to mental health in hemodialysis: ‘loss of control and displacement’, ‘lack of adjustment to and acceptance of the situation’, ‘lack of trust in the healthcare system and healthcare providers’, ‘dialysis-related symptoms, and ‘feeling overwhelmed’.

Using a co-design process,^18,19^ workshop participants identified counselling and peer support as potential strategies to address these five challenges. We performed a scoping review (manuscript in progress, unpublished) and consulted with mental health and dialysis healthcare providers and researchers, Indigenous scholars, and patient partners and determined that group DBT Skills training, which includes counselling and peer support, could help people on hemodialysis manage these challenges.

DBT was originally developed for the treatment of Borderline Personality Disorder.^20^ It has been adapted to help individuals with depression, anxiety, management of interpersonal relationships^21^ and improve adherence in adolescents on hemodialysis.^22^ DBT Skills training uses a phased approach to develop skills in mindfulness, interpersonal relationships, emotional regulation, and distress tolerance. These skills are discussed in groups and participants receive feedback from peers with similar experiences.

As noted by a Mind the Gap patient-partner with lived hemodialysis experience who is also a practising DBT therapist (RD), DBT can provide people receiving hemodialysis with many of the skills needed to manage their sense of helplessness, anger, grief, and other emotions that arise during the experience of dialysis. DBT’s unique components can be used to effectively address all five identified priority challenges to mental health. (See Quote 2, Table 1).

Our project aims to implement a 12-week group DBT Skills intervention for people who have started hemodialysis within the last 3 months or have advanced kidney disease and are likely to require hemodialysis within 6 months, and receive their kidney care at Seven Oaks General Hospital in Winnipeg. To build future program capacity and facilitate sustainability, we provided DBT Skills training (online course and co-facilitation of the DBT Skills intervention with the certified psychologist running the pilot intervention) to interested allied healthcare workers who often deal with mental health issues in people receiving hemodialysis and are already embedded within Kidney Health Manitoba (KHM). This included social workers and occupational therapists.

Our work focused on the four pillars of the Strategy for POR: implementation science; advancing Indigenous cultural competency; equity, diversity, inclusion and accessibility (EDI-A); and building capacity in patient-oriented research.

### Using Implementation Science

We used the Knowledge to Action (KTA) Framework^23^ to guide our implementation process from ‘knowledge creation’ to our current phase of ‘select, tailor and implement’ intervention; this coincides with Step 4 (Project Launch) of the Can-SOLVE CKD Network ‘Pathway to Implementation Guide.’^24^ (Figure 2).

**Figure 2.**
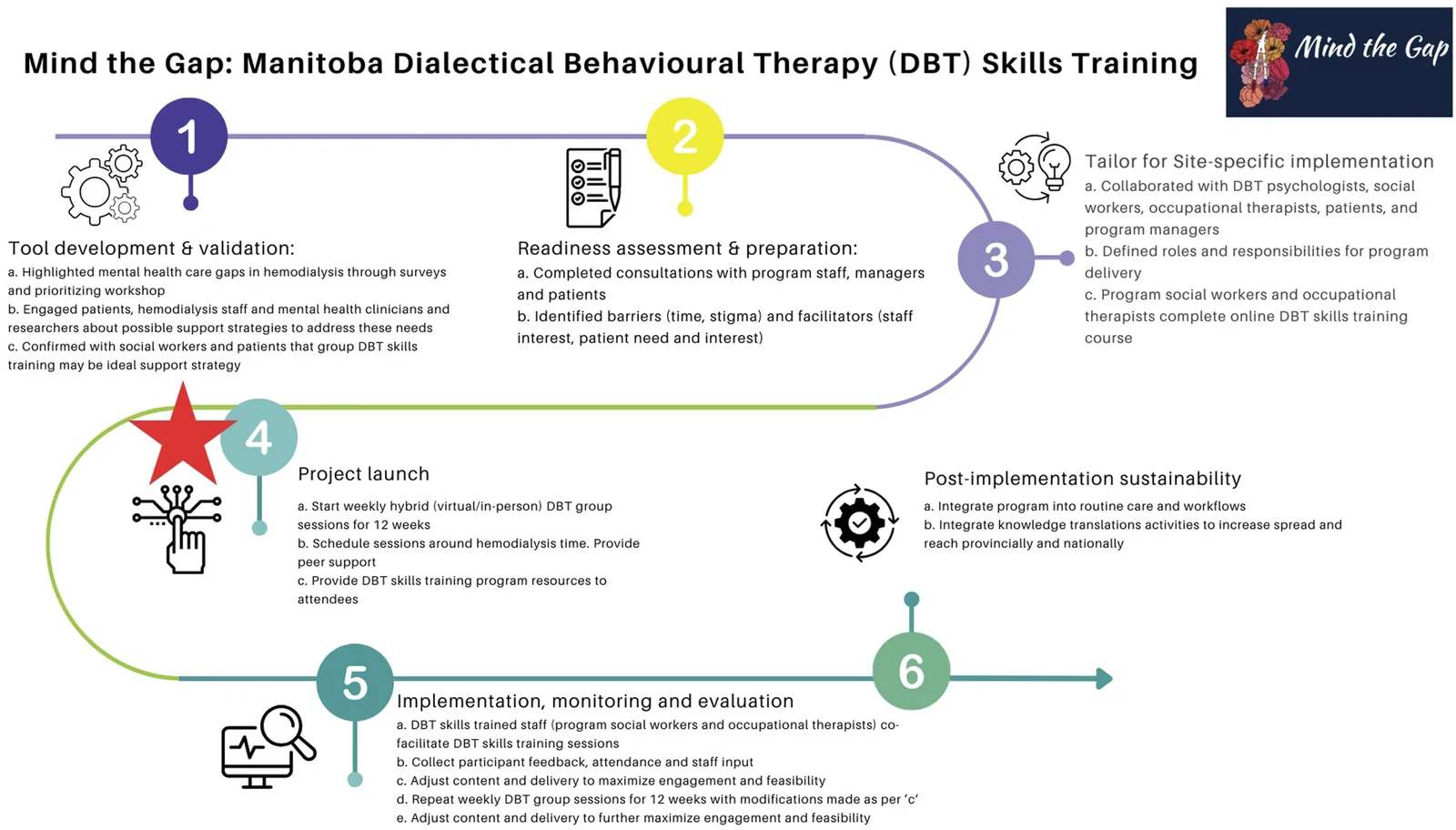
Implementation pathway for Dialectical Behavioural Therapy Skills Training Program for people receiving hemodialysis (Manitoba)

We also used the Consolidated Framework for Implementation Research (CFIR)^25^ to guide us in identifying:

a. Criteria to consider when identifying an appropriate intervention. DBT is credible, with robust evidence for effectiveness in other chronic diseases, can be piloted on a small scale and tailored to fit local context, is well designed with clear components, and is affordable within our system.
b. Barriers and enablers in the outer (SharedHealth Manitoba), inner (hospital hemodialysis unit), and person-levels (e.g. people receiving hemodialysis, hemodialysis unit staff and management) that may impact pilot implementation of the DBT Skills program in hemodialysis units in Winnipeg.
c. Key individuals and roles that are important to engage and/or include in the DBT Skills program implementation team or as intervention deliverers (i.e. patient-partners, psychologists trained in DBT, managers, social workers, and occupational therapists within KHM).
d. Strategies to address barriers to DBT implementation at person- and system-levels (e.g. conduct ongoing training, create new clinical teams, involve patients and family members and organize clinician implementation meetings).
e. Outcomes and information required to evaluate and tailor the DBT Skills Program.

### Advancing Indigenous Cultural Safety

In Canada, Indigenous people face disproportionate rates of kidney disease,^26^ as well as barriers to healthcare largely as a result of colonialism and systemic racism.^27^ It is therefore important to take meaningful steps towards Truth and Reconciliation, and particularly to address TRC Calls to Action #19 and 22, which strive to address health inequities and ensure culturally relevant care is available. To support these efforts, we ensured project leadership and core team members completed cultural safety training, and consulted Indigenous patient partners and organizations, such as the Indigenous Peoples’ Engagement and Research Council (IPERC), First Nations Health Secretariat and Social Services of Manitoba (FNHSSM), and the Manitoba Métis Federation (MMF) at various phases of the project. We engaged with a project Knowledge Keeper - important leaders within Indigenous communities – when possible and hired an Indigenous research coordinator. To date, four patient partners who are First Nations and Métis have provided guidance on our project.

### Advancing EDI-A

To help advance EDI-A, we have taken active measures to engage interest-holders with diverse roles (trainees, patient-partners, decision-makers, researchers, and healthcare providers), and different ages, life and career stages, ethnicities, and sex and gender throughout the research process. To support communication and accommodate team members, we have regularly scheduled project meetings that include check-ins with all team members. Whenever possible, we try to meet in-person and/or connect with team members through their preferred communication mode.

### Supporting Capacity Building in POR

We have taken multiple steps to ensure strong patient-partner engagement and leadership throughout all project stages, including the design of the national survey on mental health, participation in the prioritizing workshop, review of our qualitative analysis approach, data coding and interpretation of results, and selection and implementation of the DBT Skills intervention.

### Key Findings

To facilitate sustainability of the program, we have worked closely with the clinical psychology team that will provide the DBT pilot intervention and with KHM leadership to provide program social workers and occupational therapists with DBT Skills training. Discussions are ongoing to integrate DBT Skills interventions with existing workload and responsibilities. Over the past year through interest-holder consultations, we have learned of widespread enthusiasm both for improving mental health support in hemodialysis generally and specifically for the DBT Skills intervention.

### Barriers and Facilitators to Intervention Implementation

#### Barriers and Facilitators at the Person-Level

Major barriers that impede addressing mental health care at the person-level include mental health care stigma that influences whether and how patients request and access mental health supports. In our survey of mental health challenges in people receiving hemodialysis, 52% of individuals responded that they did not feel they required mental health support. Of these, 36% did not think that any action would be taken if they reported mental health challenges to their healthcare providers and 8% felt that mental health support would not be helpful.

While planning the DBT Skills intervention, we observed that Indigenous patients were more likely to participate in project activities and provide their perspective when the Mind the Gap Indigenous Research Coordinator was involved in the interactions. This speaks to the importance of ensuring team staff can provide culturally appropriate and safe care to maximize patient-partner participation.

#### Barriers and Facilitators at the Healthcare Provider-Level

Insights gathered through our national survey and while developing the DBT Skills intervention suggest that hemodialysis healthcare providers are generally supportive and recognize the need for mental health support for people receiving hemodialysis. However, due to a lack of time and training, they often feel that it is not within their scope to provide such care; a finding echoed in previous research.^3,28^ This highlights the need for increasing knowledge, potentially developing new roles or embedding trained mental health care providers into the hemodialysis unit.

Champions are a key facilitator. Overall, the Mind the Gap patient-partners have continually reinforced the importance and helped maintain momentum of this project. In Manitoba, managers, social workers, and occupational therapists within KHM have been identified as champions of the DBT Skills intervention.

#### Barriers and Facilitators to Implementation at the Intervention-Level

The DBT Skills session schedules could be a barrier and impact attendance by interfering with dialysis schedules, dialysis recovery, and life participation. We plan to address this issue by taking dialysis schedules into account when scheduling DBT sessions, providing hybrid and virtual options, and subsidizing the cost of taxi rides to attend weekly sessions for all participants. This is particularly important for a population that already struggles with reliable and flexible transportation to hemodialysis sessions.^29^ We also plan to start each session with social time and a snack to facilitate peer support and building social connections among group participants.

Concerns about the intervention’s impact on clinical care resources have been addressed by our plan to hold in-person DBT sessions in meeting rooms at Seven Oaks General Hospital, rather than in the hemodialysis unit. To support clinical program sustainability, we have provided funding and obtained approval for allied healthcare providers within KHM to complete DBT Skills training. However, in the current healthcare climate of resource restriction and cost-cutting, embedding regular delivery of DBT Skills interventions (over and above these pilot sessions) into already-overloaded work schedules and within a financially constrained healthcare system is complex and discussions are ongoing.

#### Barriers and Facilitators to Implementation at the System-Level

High turnover of decision-makers in KHM since initial conception of the DBT Skills program has removed champions and interrupted funding discussions and implementation processes.

Engagement of Indigenous scholars and mental health professionals in the DBT Skills intervention proved to be challenging. The individuals we contacted were keen to hear about the project but are so busy with pre-existing responsibilities and commitments that they were unable to participate as active team members.

Although we consulted Indigenous patient-partners at the outset of the Mind the Gap project, Indigenous engagement of entities such as MMF and FNHSSM in Manitoba happened after the project had been funded and started. Understandably, these organizations prefer to be consulted at the beginning of a project, and this has caused delays in building relationships and trust. Additionally, grant timelines need to be extended by funding agencies to allocate adequate time to engage Indigenous communities, build trust and co-develop the project.

## Objective 3b: Implement a Pathway for Mental Health Care for Albertans Receiving Dialysis

### Creating a System-Level Mental Health Pathway

In 2020, healthcare leadership from the Kidney Health Strategic Clinical Network^30^ and Alberta Kidney Care (AKC) identified mental health as a priority^31^ for Albertans receiving dialysis and requested a mental health care pathway be developed. Co-led with a Community Advisory Committee and funded by the Kidney Foundation of Canada, we developed and tailored an evidence-informed person-centred pathway for mental health care for Albertan adults receiving all forms of dialysis.^32^ The Community Advisory Committee includes nine people with lived experiences: people receiving dialysis, transplant recipients, family caregivers, and community advisors. The pathway for mental health care for people receiving dialysis was developed through literature syntheses, surveys, focus groups, interviews, and a consensus-building Delphi exercise. With this pathway developed, we co-developed Mind the Gap with the objective of informing provincial implementation of the pathway. (See Quote 3, Table 1).

### Using Implementation Science

We planned for implementation via knowledge translation - to move the mental health pathway from research into practice. We used the KTA Framework^23^ to guide our processes.

While the pathway has been developed, it has not yet been implemented (KTA Action Phase A: Identify problem). We collaboratively explored implementation with leaders in AKC-North and South in January 2024. In Fall 2024, leaders identified the Practice Direction Document (PDD) Committee as the right “home” to oversee implementation. We learned that the pathway’s 1-page flow diagram plus appendices needed to be translated into a PDD, a procedural guide for use by dialysis staff, primarily nurses. Together with two key dialysis leaders, we began to translate the pathway. This process has included numerous rounds of feedback from the PDD Committee members and Clinical Nurse Educators. The PDD will co-exist with the full pathway that will be used by all allied health and nephrologists. A patient-facing version of the pathway, “*Your journey: Coping with and adjusting to dialysis*,”^33^ was also developed through a collaborative co-design approach, that leveraged the expertise of Community Advisors as partners,^34^ and was posted online. (KTA Action Phase B: Adapt knowledge to local context).

We used the “StrategEASE tool,”^35^ to map anticipated or known barriers and facilitators to change strategies (KTA Action Phase C: Access barriers to knowledge use). The strategies we will leverage include the use of site champions, leaders to support change, audit and feedback loops, education with resources, and dissemination of the patient-facing version of the pathway.

The PDD and pathway will be introduced in two dialysis sites, one in AKC-North and the other in AKC-South, to assess contextual factors influencing future provincial implementation. This coincides with Step 4 (Project Launch) of the Can-SOLVE CKD Network ‘Pathway to Implementation Guide.’^24^ (Figure 3). Findings will inform refinement of the PDD and pathway, implementation strategies, and recommendations for provincial implementation (KTA Action Phase D: Tailor and implement intervention).

**Figure 3.**
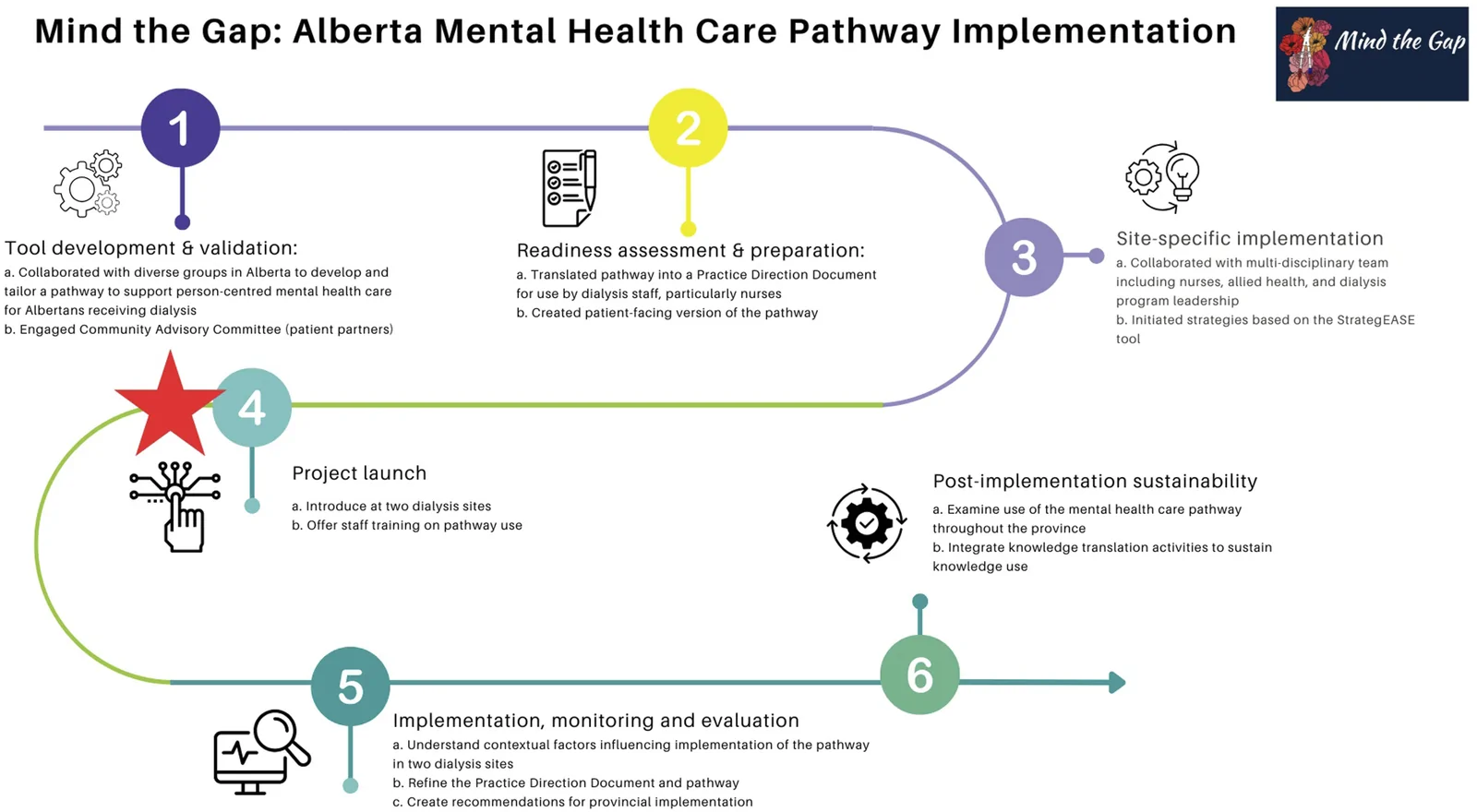
Implementation pathway for Provincial Pathway for Mental Health Care for People Receiving Dialysis (Alberta)

To further understand how to effectively and strategically engage in knowledge translation – we also drew upon:

a) the Consolidated Framework for Implementation Research (CFIR)^25^ to assess contextual factors that would influence implementation at a health-system level. Leveraging Rogers’ Diffusion of Innovation,^36^ its strength lies in its application to healthcare systems.
b) the Theoretical Domains Framework (TDF)^37^ to acknowledge behavior change necessary with implementation, and to map implementation interventions onto barriers and facilitators. This framework is appropriate because attending to mental health in dialysis settings has not been traditional practice, may be counter-culture, and considered by many kidney healthcare providers to be outside of their role.^3,32^

### Advancing EDI-A

EDI-A principles were incorporated into implementation efforts in numerous ways. Notably, the Community Advisory group included diverse people with lived experience, and intentionally included under-represented perspectives (e.g., age, gender, cultural background, geography, experiences with mental health). We strove to find Community Advisory meeting times that worked best for everyone, held in-person meetings when possible, and dedicated time to listen to the lived experience of patient partners. We collaborated on patient-facing materials. We intentionally used language that did not perpetuate stigma pertaining to mental health. For example, the Advisors suggested that instead of using “mental health” in the title of the patient-facing pathway, “coping with and adjusting to dialysis” would be more easily understood and welcomed. It was also essential to set boundaries on the scope of the pathway. For example, the pathway does not address pre-dialysis, pediatrics, conservative care, family caregivers, or Indigenous ways of knowing and healing. Rather, each requires intentional, patient partner-guided focused inquiry.

### Supporting Capacity Building in POR

In collaboration, all aspects of planning for implementation of the pathway have been co-led with the established Community Advisory Committee.

### Key Findings

Transitioning from a research-driven to quality improvement-driven initiative meant shifting from knowledge generation to application at a system-level. Collaboration with existing (and shifting) dialysis operations structures, such as the PDD Committee, was critical to move the pathway from research into practice.

### Barriers and Facilitators to Intervention Implementation

#### Barriers and Facilitators at the Person-Level

A common barrier is the belief that mental health support should only be provided outside of dialysis care. Healthcare is traditionally organized by medical specialty and providers have become accustomed to providing siloed care, which poses challenges to continuity of care and person-centred care.

Recognizing these barriers, the online patient-facing version of the pathway^33,34^ may be used as a facilitator to empower people receiving dialysis to advocate for their mental health care needs. The Alberta Health Services Provincial Pathways Unit^38^ collaborated on this co-design; provided a template; helped with graphics, peer-review, website creation; and co-authored the publication. They will provide routine analytics on website “clicks”, monitoring the use of the provincial pathway for mental health care for people receiving dialysis.

#### Barriers and Facilitators at the Healthcare Provider-Level

During discussions with kidney healthcare providers working towards implementation of the provincial pathway for mental health care for people receiving dialysis, we heard that this was a “difficult topic.” Healthcare providers are not immune to the stigma of engagement in providing mental health care. They often said that they did not know how to start the conversation, a comment echoed by patients (see Quote 4, Table 1).

Champions are a strategic facilitator. The provincial pathway for mental health care for people receiving dialysis also had ongoing champions within its established Steering Committee, a decision-making body including provincial decision-makers, as well as representation from the Community Advisory Committee.^32^ New AKC champions in dialysis operations, care managers, and Clinical Nurse Educators emerged during the translational work to create a PDD for nurses.

#### Barriers and Facilitators to Implementation at the Intervention-Level

One facilitator for the for the provincial pathway for mental health care for people receiving dialysis was the many knowledge users from AKC who participated in developing and tailoring the pathway for mental health care. One of the key barriers both at an intervention and system-level has been ownership: transitioning from a research project to a quality improvement project embedded in the health care system. While quality improvement initiatives are continuous throughout the province, there is no dedicated group with a primary function on quality improvement to oversee clinical implementation of the pathway.

#### Barriers and Facilitators to Implementation at the System-Level

Turnover of senior program leadership in AKC has significantly delayed implementation strategies for the provincial pathway for mental health care for people receiving dialysis.

## Limitations

Our work reflects the perspectives of interest-holders in two Canadian provinces. Transferability of findings about mental healthcare challenges, available supports, and interventions to diverse dialysis populations across Canadian contexts requires further investigation.

## Conclusion

The need to attend to mental health support for people receiving dialysis is evident. The process involves numerous challenges, including stigma regarding patients accessing or healthcare providers engaging in mental health supports, time constraints among staff and inflexible, siloed structures within healthcare systems. [See Quote 5, Table 1] Nevertheless, our findings suggest that this work is valued by interest-holders, and there are opportunities to identify champions and tailor tools for meaningful support. Blending mental health care with dialysis care is culturally novel and an important step to meaningfully support the emotional and mental well-being of people on dialysis. [See Quote, 6 Table 1].

## ORCID iDs

Kara Schick-Makaroff https://orcid.org/0000-0001-6200-3416

Mark Canney https://orcid.org/0000-0002-4308-3083

Oksana Harasemiw https://orcid.org/0000-0001-9747-5632

Scott Klarenbach https://orcid.org/0000-0002-8611-1056

Jonathan Lee https://orcid.org/0009-0005-3273-8741

Primrose Mharapara https://orcid.org/0009-0009-6691-4239

Richard Sawatzky https://orcid.org/0000-0002-8042-190X

Karthik Tennankore https://orcid.org/0000-0002-7919-6709

Stephanie Thompson https://orcid.org/0000-0003-3109-6837

Clara Bohm https://orcid.org/0000-0001-7710-7162

## References

[1] World Health Organization: WHO . Depressive disorder (depression). 2025. https://www.who.int/news-room/fact-sheets/detail/depression

[2] Palmer S Vecchio M Craig JC , et al. Prevalence of depression in chronic kidney disease: systematic review and meta-analysis of observational studies. Kidney International. 2013;84(1):179-191. doi:10.1038/ki.2013.77. 23486521

[3] Schick-Makaroff K Wozniak LA Short H , et al. Burden of mental health symptoms and perceptions of their management in in-centre hemodialysis care: a mixed methods study. J Patient Rep Outcomes. 2021;5:111. doi:10.1186/s41687-021-00385-z. PMC8555046 34709470

[4] Cukor D Coplan J Brown C , et al. Depression and anxiety in urban hemodialysis patients. Clinical Journal of the American Society of Nephrology. 2007;2(3):484-490. doi:10.2215/cjn.00040107. 17699455

[5] Abbas TS Ebrahimnia M Shamspour N Assari S . Effect of depression on health care utilization in patients with end-stage renal disease treated with hemodialysis. Eur J Intern Med. 2009;20(4):411-414. 10.1016/j.ejim.2009.03.007 19524185

[6] Lopes AA Bragg J Young E Dialysis Outcomes and Practice Patterns Study DOPPS , et al. Depression as a predictor of mortality and hospitalization among hemodialysis patients in the United States and Europe. Kidney Int. 2002;62(1):199-207. 10.1046/j.1523-1755.2002.00411.x 12081579

[7] Hedayati SS Minhajuddin AT Afshar M Toto RD Trivedi MH Rush AJ . Association between major depressive episodes in patients with chronic kidney disease and initiation of dialysis, hospitalization, or death. J Am Med Assoc. 2010;303(19):1946-1953. 10.1001/jama.2010.619 PMC3217259 20483971

[8] Chan L Tummalapalli SL Ferrandino R , et al. The effect of depression in chronic hemodialysis patients on inpatient hospitalization outcomes. Blood Purif. 2017;43(1-3):226-234. 10.1159/000452750 PMC5823516 28114133

[9] Farrokhi F Abedi N Beyene J Kurdyak P Jassal SA . Association between depression and mortality in patients receiving long-term dialysis: A systematic review and meta-analysis. Am J Kidney Dis. 2014;63(4):623-635. 10.1053/j.ajkd.2013.08.024 24183836

[10] Knight EL Ofsthun N Teng M Lazarus JM Curhan GC . The association between mental health, physical function, and hemodialysis mortality. Kidney Int. 2003;63(5):1843-1851. doi:10.1046/j.1523-1755.2003.00931.x. PMID: 12675862. 12675862

[11] Szeifert L Bragg-Gresham J Thumma J , et al. Psychosocial variables are associated with being wait-listed, but not with receiving a kidney transplant in the Dialysis Outcomes and Practice Patterns Study (DOPPS). Nephrology Dialysis Transplantation. 2012;27(5):2107-2113. 10.1093/ndt/gfr568 22058174

[12] Fernandez L Thompson S Berendonk C Schick-Makaroff K . Mental health care for adults treated with dialysis in Canada: A scoping review. Canadian Journal of Kidney Health and Disease. 2022;9(1):1-16. doi:10.1177/20543581221086328. PMC8950027 35340771

[13] Rossum K Finlay J McCormick M , et al. A Mixed Method Investigation to Determine Priorities for Improving Information, Interaction, and Individualization of Care Among Individuals on In-center Hemodialysis: The Triple I Study. Can J Kidney Health Dis. 2020;7:2054358120953284. doi:10.1177/2054358120953284. PMC7580147 33149921

[14] Levin A Adams E Barrett BJ , et al. Canadians seeking solutions and innovations to overcome chronic kidney disease (Can-SOLVE CKD): form and function. Canadian journal of kidney health and disease. 2018;5:2054358117749530. 10.1177/2054358117749530 PMC5774731 29372064

[15] Healthyqol.com. (n.d.). https://www.healthyqol.com/kidney

[16] Mharapara P Olson J Ahmed SB Schick-Makaroff K . Nursing knowledge in the care of patients on hemodialysis who are living with depression: An integrative review. Nephrology Nursing Journal. 2025;52(3):265-286. https://pubmed.ncbi.nlm.nih.gov/40623233/40623233

[17] Mharapara P Ahmed SB Olson J Schick-Makaroff K . Sex and gender characteristics of adults on hemodialysis experiencing depression and depressive symptoms: A scoping review. Canadian Journal of Kidney Health and Disease. 2025;12:1-8. doi:10.1111/hex.70466 PMC12536104 41122301

[18] Chinaleong-Brooks K . Understanding co-design [Internet]. Alberta Health Services. Strategic Clinical Networks TM Patient and Family Engagement Team; 2020. [cited 2026 Feb 24]. Available from. https://www.albertahealthservices.ca/assets/about/scn/ahs-scn-pfe-understanding-codesign.pdfIdolikethesoc

[19] Co-design toolkit. NSW Government. [Internet][cited 2026 Feb 24]. Available from. https://aci.health.nsw.gov.au/projects/co-design/step-by-step

[20] May JM Richardi TM Barth KS . Dialectical behavior therapy as treatment for borderline personality disorder. Ment Health Clin. 2016;6(2):62-67. doi:10.9740/mhc.2016.03.62. PMID: 29955449; PMCID: PMC6007584. PMC6007584 29955449

[21] Corliss J . Dialectical Behavioural Therapy: What is it and who can it help? Harvard Health Publishing. Harvard Medical School. 2024. https://www.health.harvard.edu/blog/dialectical-behavior-therapy-what-is-it-and-who-can-it-help-202401223009. Accessed Oct 19, 2025.

[22] Hashim BL Vadnais M Miller AL . Improving Adherence in Adolescent Chronic Kidney Disease: A Dialectical Behavior Therapy (DBT) Feasibility Trial. Clinical Practice in Pediatric Psychology. 2013;1(4):369-379. doi:10.1037/cpp0000024.

[23] Graham I Logan J Harrison M , et al. Lost in knowledge translation: time for a map? J Contin Educ Health Prof. 2006;26:13-24. doi:10.1002/chp.47. 16557505

[24] Pathway to Implementation A Six-Step Guide for Implementing an Evidence-Based Health Intervention. Can-SOLVE CKD. 2023. [Internet] [cited 2025 Dec 18]. Available from. https://cansolveckd.ca/wp-content/uploads/2023/10/Pathway-to-Implementation-2023-09-25.pdf

[25] Damschroder LJ Reardon CM Widerquist MAO Lowery J . The updated Consolidated Framework for Implementation Research based on user feedback. Implementation Science. 2022;17(1):75. doi:10.1186/s13012-022-01245-0. PMC9617234 36309746

[26] Laurier N Stoesz T Quaiattini A , et al. Examining inequities in clinical outcomes for Indigenous patients treated with dialysis in Canada: A scoping review. Kidney Medicine [Internet]. 2025;7(11):101106. Available from: doi:10.1016/j.xkme.2025.101106. PMC12597283 41216358

[27] Allan B Smylie J . First Peoples, Second Class Treatment: The Role of Racism in the Health and Well-being of Indigenous Peoples in Canada; 2015.

[28] Lehecka A Mendelssohn D Hercz G . Nephrologists’ Attitudes Regarding Psychosocial Care in Hemodialysis Units. Canadian Journal of Kidney Health and Disease. 2021;8:20543581211037426. doi:10.1177/20543581211037426. PMC8361505 34394946

[29] Lewis RA Bohm C Fraser F , et al. Transportation burden associated with hemodialysis in Canada: A Qualitative study of Stakeholders. Kidney Medicine [Internet]. Kidney Med. 2022;5(2):100571. Available from: doi:10.1016/j.xkme.2022.100571. PMC9851886 36686595

[30] Pannu N Gilmour L Klarenbach S Kidney Health Strategic Clinical Network . Kidney Health Strategic Clinical Network. Kidney health strategic clinical Network: driving positive change to optimize kidney health in Alberta. Can Med Assoc J. 2019;191(Suppl):S39-S41. doi:10.1503/cmaj.190573. PMC6901194 31801764

[31] Transformational Roadmap 2021-2026 . Medicine Strategic Clinic Network; 2022. Retrieved October 23, 2025, from. https://www.albertahealthservices.ca/assets/about/scn/ahs-scn-med-trm.pdf

[32] Schick-Makaroff K Berendonk C Salum M , et al. Developing and tailoring a person-centred pathway for mental health care for people receiving dialysis. Nephron. 2025;149:392-410. doi:10.1159/000544058. PMC12215154 39956103

[33] Coping With and Adjusting to Dialysis: Emotional well-being and dialysis. (n.d). https://myhealth.alberta.ca/HealthTopics/coping-and-adjusting-to-dialysis-pathway

[34] Albers A Berendonk C Robison J , et al. Co-Designing a Patient-Facing Version of a Mental Healthcare Pathway for People Receiving Dialysis. Patient Experience Journal. 2063;12(3):233-245. doi:10.35680/2372-0247.

[35] Free StrategEase tool — The Center for Implementation . The Center for Implementation; n.d. https://thecenterforimplementation.com/strategease-tool

[36] Rogers EM . Diffusion of Innovations. 5th edition New York, NY: Free Press; 2003.

[37] Atkins L Francis J Islam R , et al. A guide to using the Theoretical Domains Framework of behaviour change to investigate implementation problems. Implementation Science. 2017;12(1):77. doi:10.1186/s13012-017-0605-9. PMC5480145 28637486

[38] Alberta Health Services . About Provincial Pathways Unit | Alberta Health Services. 2026. https://www.albertahealthservices.ca/aph/Page18461.aspx

